# Loss of mTOR repressors Tsc1 or Pten has divergent effects on excitatory and inhibitory synaptic transmission in single hippocampal neuron cultures

**DOI:** 10.3389/fnmol.2014.00001

**Published:** 2014-02-03

**Authors:** Matthew C. Weston, Hongmei Chen, John W. Swann

**Affiliations:** ^1^The Cain Foundation Laboratories, The Jan and Dan Duncan Neurological Research InstituteHouston, TX, USA; ^2^Departments of Neuroscience and Pediatrics, Baylor College of MedicineHouston, TX, USA

**Keywords:** pten, TSC/mTOR, synaptic transmission, akt, excitatory transmission, inhibitory transmission, epilepsy, autism

## Abstract

The *Pten* and *Tsc1* genes both encode proteins that repress mechanistic target of rapamycin (mTOR) signaling. Disruption of either gene in the brain results in epilepsy and autism-like symptoms in humans and mouse models, therefore it is important to understand the molecular and physiological events that lead from gene disruption to disease phenotypes. Given the similar roles these two molecules play in the regulation of cellular growth and the overlap in the phenotypes that result from their loss, we predicted that the deletion of either the *Pten* or *Tsc1* gene from autaptic hippocampal neurons would have similar effects on neuronal morphology and synaptic transmission. Accordingly, we found that loss of either Pten or Tsc1 caused comparable increases in soma size, dendrite length and action potential properties. However, the effects of Pten and Tsc1 loss on synaptic transmission were different. Loss of Pten lead to an increase in both excitatory and inhibitory neurotransmission, while loss of Tsc1 did not affect excitatory neurotransmission and reduced inhibitory transmission by decreasing mIPSC amplitude. Although the loss of Pten or Tsc1 both increased downstream mTORC1 signaling, phosphorylation of Akt was increased in Pten-ko and decreased in Tsc1-ko neurons, potentially accounting for the different effects on synaptic transmission. Despite the different effects at the synaptic level, our data suggest that loss of Pten or Tsc1 may both lead to an increase in the ratio of excitation to inhibition at the network level, an effect that has been proposed to underlie both epilepsy and autism.

## INTRODUCTION

The evolutionarily conserved mTOR signaling network integrates information from growth factor receptor activity, ATP, glucose, and amino acid levels to control cellular growth, proliferation, and autophagy (Sarbassov et al., 2005a; Wullschleger et al., 2006; Laplante and Sabatini, 2012). In line with these essential cellular functions, mTOR dysfunction is associated with a broad range of human diseases including cancer, type II diabetes and obesity, and major efforts to develop drugs targeting the pathway are underway (Guertin and Sabatini, 2009). In the brain, hyperactivation of mTOR signaling causes neurological disorders in both humans and in animal models. Although dependent on the mutation, the region affected and the extent of expression, the most common symptoms are epilepsy, autism, and intellectual disability (Swiech et al., 2008; Ehninger et al., 2009; Hoeffer and Klann, 2009; Russo et al., 2012).

Two established genetic mechanisms through which mTOR signaling abnormalities lead to neurological disease are mutations that disrupt the *Pten* or *Tsc1* genes. The protein products of these genes are similar in many ways: (1) they are both repressors of mTOR signaling and in their absence the mTORC1 complex is hyperactive, (2) their loss in neurons can lead to hyperexcitable network activity, and (3) rapamycin, an inhibitor of mTOR, blocks the cellular and behavioral consequences of their loss in animal models, and potentially humans as well (Krueger et al., 2013). This evidence predicts that the effects of Pten or Tsc1/2 disruption on neuronal form and function may be similar.

At the molecular level, studies from yeast and in mammalian cell lines, mostly in the context of cancer research, have provided a wealth of information on the basic organization of the mTOR pathway and the roles that Pten and Tsc1 play. From these studies it is clear that one of the major inputs to mTOR activity is insulin and growth factor receptor signaling (Harris and Lawrence, 2003; Manning and Cantley, 2003; Avruch et al., 2006; Howell and Manning, 2011). Receptor activation, through a series of intracellular events utilizing phosphoinositide-3 kinase (PI3K), leads to increased PIP_3_ levels and membrane localization of Akt, a state that favors its phosphorylation. Pten represses this pathway by catalyzing the conversion of PIP_3_ to PIP_2_, which ultimately leads to reduced Akt phosphorylation and reduced activity of Akt toward its substrates. One important substrate of Akt is Tsc2, which complexes with Tsc1 to function as a GTPase activating protein that, via Rheb, inhibits mTORC1. Loss of either Pten or Tsc1 are both thought to disrupt these molecular events and lead to excessive activity of mTORC1, however, the unique position of each protein in the pathway and the potential of each to act on targets other than their canonical mTOR-related ones raise the possibility that the functional consequences of their loss may not be overlapping.

In neurons, previous studies have consistently shown that loss of either Pten or Tsc1/2 increases neuronal cell body size (Backman et al., 2001; Kwon et al., 2001; Tavazoie et al., 2005; Meikle et al., 2007; Feliciano et al., 2011). Loss or reduction of Pten also increases growth of axons, dendrites, and spine/synapse formation (Jaworski et al., 2005; Kwon et al., 2006; Fraser et al., 2008). While loss of Tsc1/2 has been shown to increase axon growth and dendritic complexity (Meikle et al., 2007; Choi et al., 2008; Feliciano et al., 2011, 2012), the reported effects on spine and synapse number are variable (Tavazoie et al., 2005; Meikle et al., 2007; Bateup et al., 2011; Tsai et al., 2012). From a physiological standpoint, loss of Pten has been shown to increase excitatory synaptic transmission due to increases in the mEPSC amplitude (Luikart et al., 2011; Weston et al., 2012; Xiong et al., 2012). While loss of Tsc1 has also been linked to increased excitation, the underlying mechanism is not clear (Tavazoie et al., 2005; Wang et al., 2007; Bateup et al., 2011, 2013). Differences in neuron type, time, and extent of gene deletion and secondary effects of seizures may all have contributed to these variable results.

In order to test the extent to which loss of Pten or Tsc1 has overlapping effects on neuronal form and function, we performed a direct comparison of the cellular neurophysiology and morphology of autaptic hippocampal neurons in which *Pten* or *Tsc1* was genetically disrupted. We found that loss of Pten or Tsc1 caused similar changes in neuronal morphology, passive membrane, and action potential properties and downstream mTORC1 signaling. The effects of loss of Pten or Tsc1 on synaptic transmission were, however, different. Loss of Pten increased excitatory neurotransmission, while loss of Tsc1 had no effect. Loss of Pten also increased inhibitory synaptic transmission, while loss of Tsc1 decreased it, suggesting that, despite their many similarities, loss of Pten and Tsc1 may lead to a disruption of the excitatory-inhibitory balance in the hippocampus through distinct mechanisms.

## RESULTS

In order to directly compare the cellular effects of Pten or Tsc1 loss, we made primary cultures of hippocampal neurons from either Pten^loxP/loxP^ or Tsc1^loxP/loxP^ mice and infected them with a lentivirus expressing a Cre recombinase-RFP fusion protein (hereafter referred to as Pten-ko and Tsc1-ko neurons). Control neurons were either loxP/loxP neurons from the same animal infected with a control virus or wild-type littermates infected with the Cre virus. The cultures were single-neuron (autaptic) cultures on astrocyte islands. This preparation allowed us to simultaneously test both pre- and postsynaptic function, to quantify several electrophysiological and morphological parameters on a per cell basis, and to examine the effects of Pten and Tsc1 loss in the absence of network or activity-dependent effects.

**FIGURE 1:**
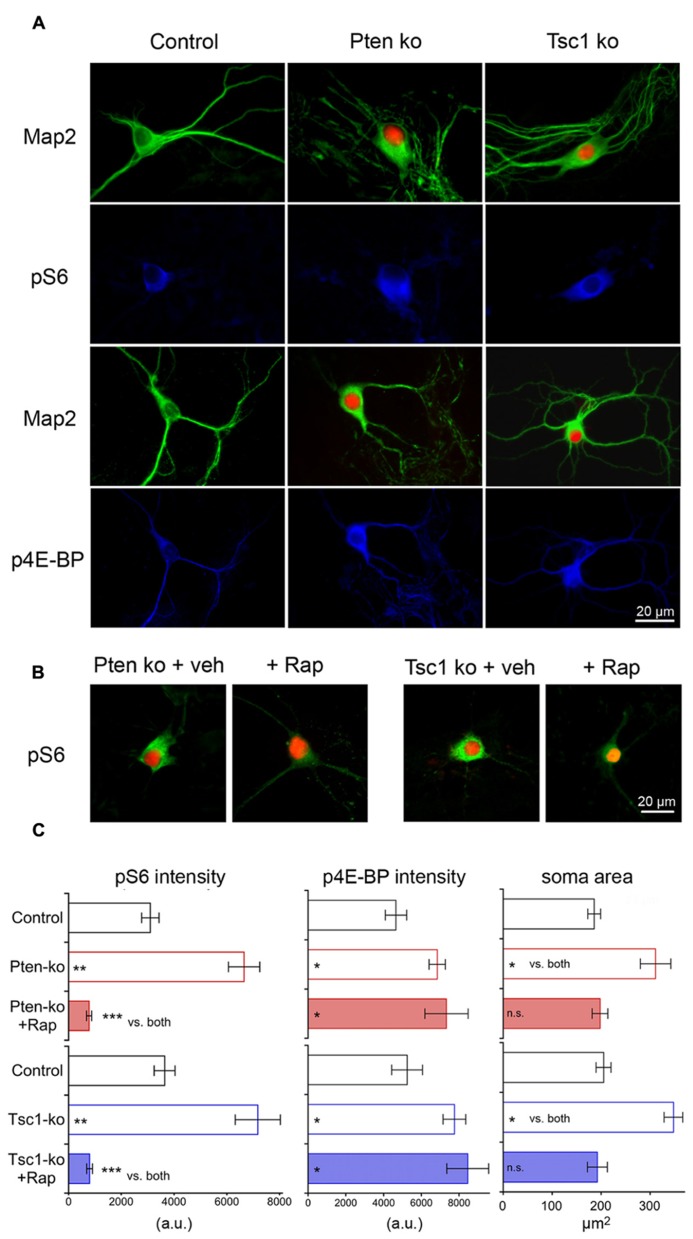
**Cre-mediated deletion of Pten or Tsc1 causes mTORC1 activation and an increase in cell size. (A)** Representative images showing immunofluorescence from Map2 (green) and either phospho-S6 or phospho-4E-BP (blue) from control (left column), Pten-ko (middle column) and Tsc1-ko (right column) neurons. Red fluorescence is shown in Pten- and Tsc1-ko neurons from the Cre-RFP fusion protein. **(B)** Representative images showing immunofluorescence from phospho-S6 (green) and the Cre-RFP fusion protein from Pten-ko (left images) and Tsc1-ko (right images) treated with either DMSO vehicle or 20 nM rapamycin for 72 h. **(C)** Summary data showing the changes in pS6, p4E-BP and soma area (mean ± SEM) for control, knockout and rapamycin-treated knockout neurons for Pten (red bars) and Tsc1 (blue bars). ^*^p ≤ 0.05, ^*^^*^p ≤ 0.01, ^*^^*^^*^p ≤ 0.001, n.s. = not significant vs. control unless indicated.

We verified that the cre-mediated gene deletion led to expected increases in mTOR signaling by testing for increased levels of phospho-S6 (pS6) and phospho-4E binding protein (p4E-BP), as well as increased soma size, which are three well-characterized consequences of hyperactive mTOR signaling (**Figure 1**). Using quantitative immunofluorescence analysis of both Pten- and Tsc1- deficient single neurons (**Figure 1A**), we found that the loss of either Pten or Tsc1 caused significant increases in both pS6 and p4E-BP immunofluorescence intensity relative to controls (**Figure 1C**). We also immunostained for Map2, to visualize the soma of Pten- and Tsc1-ko neurons. We then imaged the neurons, traced the cell bodies using Neurolucida software and measured their area. The area of the cell body was significantly increased in both Pten- and Tsc-ko neurons (**Figure 1C**). Finally, to verify that the changes in pS6 levels and soma size were due to hyperactive mTOR in both mutants, we treated the neurons with 20 nM rapamycin at DIV 7 for 72 h and repeated the pS6, p4E-BP, and cell size measurements. Rapamycin treatment reduced both pS6 levels and soma area in both Pten and Tsc1-ko neurons to control levels or lower (**Figures 1B,C**). p4E-BP levels in Pten and Tsc1-ko neurons were not decreased by rapamycin treatment (**Figure 1C**), in agreement with previously reported effects of long-term rapamycin treatment on 4E-BP phosphorylation in some cell types (Choo et al., 2008; Thoreen et al., 2009).

### LOSS OF Pten OR Tsc1 AFFECTS PASSIVE MEMBRANE AND ACTION POTENTIAL PROPERTIES

**FIGURE 2:**
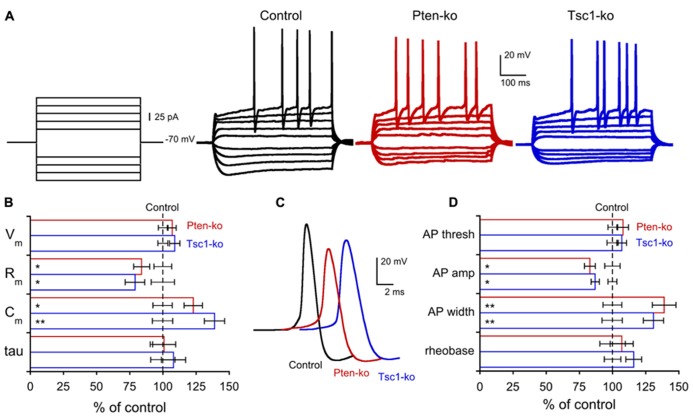
**Pten- and Tsc1 knockout neurons show similar alterations in passive membrane and action potential properties. (A)** Representative responses of control (black traces), Pten-ko (red traces), and Tsc1-ko (blue traces) neurons to the current injection protocol indicated at left. **(B)** Values (mean ± SEM) for resting membrane potential (Vm), input resistance (Rm), membrane capacitance (Cm), and membrane time constant (tau) for Pten-ko (red bars) and Tsc1-ko (blue bars) relative to their respective controls (black dashed line). **(C)** Example traces of action potentials evoked by current injection to control (black trace), Pten-ko (red trace), and Tsc1-ko (blue trace) neurons. **(D)** Mean ± SEM values for action potential (AP) threshold, peak amplitude, and half-width, as well as the amount of current injected to elicit an AP (rheobase), for Pten-ko (red bars), and Tsc1-ko (blue bars) relative to their respective controls (black dashed line). ^*^p ≤ 0.05, ^*^^*^p ≤ 0.01 vs. control

Previous electrophysiological studies of both Pten and Tsc-lacking neurons have consistently shown effects on passive membrane properties, especially membrane capacitance, consistent with the large influence that the mTOR signaling pathway has on regulation of cellular growth (Bateup et al., 2011, 2013; Luikart et al., 2011; Weston et al., 2012; Xiong et al., 2012; Normand et al., 2013). In order to verify this effect in our system, and to compare the magnitude of the changes induced by a loss of Pten or Tsc1, we performed a whole cell patch current clamp analysis of the neurons (**Figure 2**; **Table 1**). The resting membrane potentials (*V*_m_) of Pten- and Tsc1-ko neurons were not different from their respective controls (**Figure 2B**). Injection of hyperpolarizing current steps and then measuring the time course and extent of the resulting voltage changes revealed that neither Pten- nor Tsc1-ko neurons had altered membrane time constants, but that the input resistance was significantly decreased to similar levels for both knockouts (**Figure 2B**). As a result, the calculated membrane capacitance was also increased to a similar extent for both Pten- and Tsc1-ko neurons vs. control (**Figure 2B**).

**Table 1:** Passive membrane and AP properties of Pten- and Tsc1-ko neurons.

	Pten		Tsc1	
	Control, *n* = 32	Pten-ko, *n* = 34	Control, *n* = 33	Tsc1-ko, *n* = 36
*V*_(rest)_ (mV)	-58.0 ± 2.2	-62.1 ± 1.9	-59.7 ± 2.1	-63.1 ± 2.4
*R*_(input)_ (MΩ)	438 ± 31	359 ± 26^*^	390 ± 36	302 ± 26^*^
Time constant (ms)	34.2 ± 3.3	34.1 ± 2.9	32.3 ± 2.5	34.8 ± 2.6
C_m_ (pF)	81.3 ± 5.7	99.6 ± 7.0^*^	79.5 ± 5.7	108.0 ± 8.5^*^^*^
AP threshold (mV)	-29.1 ± 1.0	-31.9 ± 1.3	-32.9 ± 1.2	-35.6 ± 1.3
AP amplitude (mV)	88.4 ± 5.2	74.3 ± 3.0^*^	66.7 ± 2.8	57.6 ± 1.9^*^
AP half-width (ms)	1.16 ± 0.08	1.61 ± 0.14^*^^*^	1.26 ± 0.09	1.58 ± 0.12^*^
Current to first AP (pA)	176 ± 17	189 ± 12	155 ± 13	177 ± 9

Measurements are shown as mean ± SEM; ^*^p ≤ 0.05, ^*^^*^p ≤ 0.01 compared to control with unpaired *t*-test or Mann–Whitney.

Next, we injected depolarizing current steps to examine the action potential (AP) properties of Pten- and Tsc1-ko neurons. The AP threshold was not different in either Pten- or Tsc1-ko neurons, however, there were significant effects on the shape of the AP in both Pten- and Tsc1-ko neurons (**Figure 2C**). Both mutants had action potentials that were smaller in amplitude and with a greater duration at half the maximal amplitude (AP half-width) than their controls (**Figures 2C,D**). Despite the reduced input resistance, there was no difference in the amount of injected current needed to elicit an AP (**Figure 2D**), or the number of APs for a given current injection (**Figure 2A**).

### LOSS OF Pten INCREASES AP EVOKED GLUTAMATERGIC TRANSMISSION BUT LOSS OF Tsc1 DOES NOT

**FIGURE 3:**
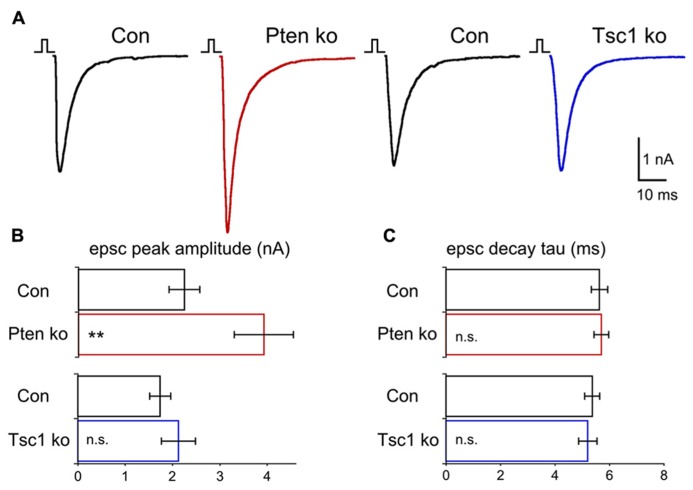
**Pten-ko neurons show increased AP-evoked EPSCs but Tsc1-ko neurons do not. (A)** Representative responses of voltage-clamped Pten-ko (red trace) and Tsc1-ko (blue traces) neurons and their controls (black traces) to a 2 ms depolarization to 0 mV from -70 mV. **(B)** Bar graph showing AP-evoked EPSC peak amplitudes (mean ± SEM) from Pten-ko (red bars) and Tsc1-ko (blue bars) and their respective controls (black bars). **(C)** Mean ± SEM values for AP-evoked EPSC decay time constant from Pten-ko (red bars) and Tsc1-ko (blue bars) and their respective controls (black bars). ^*^^*^p ≤ 0.01, n.s. = not significant.

Previous studies of both Pten- and Tsc1-ko mice have suggested that altered excitatory transmission in these models is an important contributor to the disease phenotypes (Wang et al., 2007; Luikart et al., 2011; Xiong et al., 2012). We compared the effects of Pten and Tsc1 loss on excitatory synaptic transmission using whole cell voltage clamp analysis of glutamatergic neurons. We began by evoking action potentials with a 2 ms depolarization to 0 mV and recording the resulting EPSCs. Blockade of the EPSCs with 300 mM kynurenic acid confirmed they were glutamatergic. In Pten-ko neurons, peak EPSC amplitudes were increased 70% relative to control neurons (**Figures 3A,B**). The peak EPSC amplitudes of Tsc1-ko neurons, however, were not different from control neurons (**Figures 3A,B**). The shape of the EPSCs in both Pten- and Tsc1-ko neurons, as measured by the rise and decay time constants, were unchanged relative to control (**Figure 3C**), indicating that the synchrony of vesicle release and the AMPA receptor subunit composition may be unchanged.

### LOSS OF Pten INCREASES MINIATURE EPSC TRANSMISSION BUT LOSS OF Tsc1 DOES NOT

In theory, changes in several parameters could account for the increase in AP-evoked EPSC amplitude in Pten-ko neurons. These include: (1) miniature EPSC (mEPSC) amplitude, (2) total number of synapses or releasable synaptic vesicles, or (3) probability of vesicle release (*P*_vr_). Even in the Tsc1-ko neurons, increases in one of these parameters may be offset by a decrease in another. We therefore attempted to determine the contribution of each parameter to excitatory synaptic transmission in the Pten- and Tsc1-ko neurons.

**FIGURE 4:**
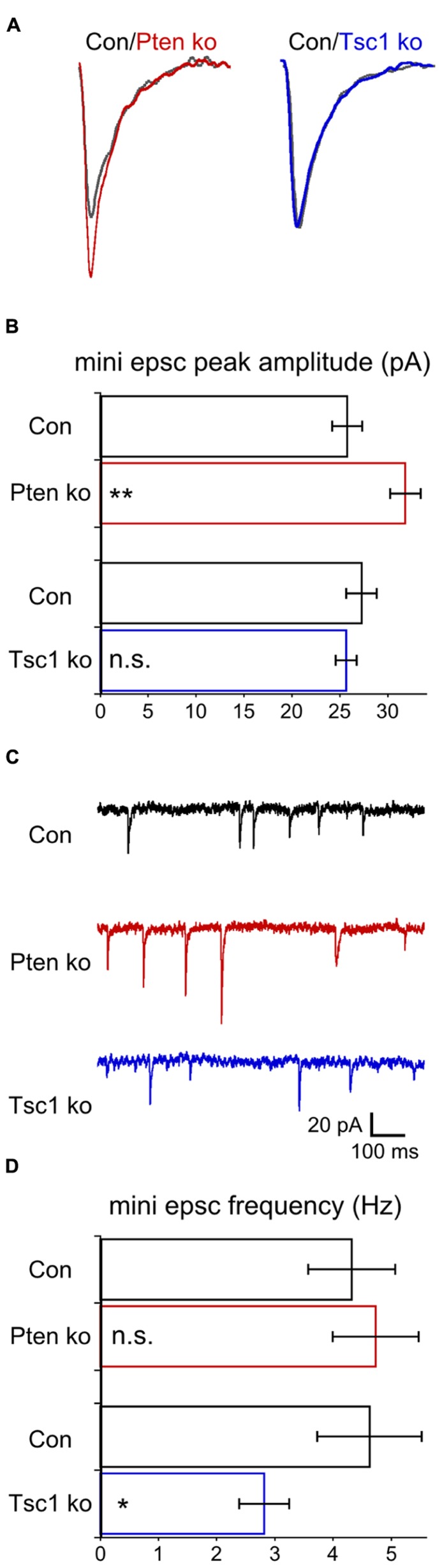
**Pten-ko neurons show increased mEPSC amplitude but Tsc1-ko neurons do not. (A)** Representative traces showing the average of mEPSCs collected from one Pten-ko neuron (red trace), one Tsc1-ko neuron (blue trace), and their respective controls (overlaid gray traces). **(B)** Bar graph showing mEPSC peak amplitude (mean ± SEM) of Pten-ko (red bars) and Tsc1-ko (blue bars) neurons and their respective controls (black bars). **(C)** Representative traces showing control (black trace), Pten-ko (red trace), and Tsc1-ko (blue trace) miniature postsynaptic current activity from hippocampal glutamatergic neurons. **(D)** Bar graph showing mEPSC frequency values (mean ± SEM) in Hz for Pten-ko (red bars) and Tsc1-ko (blue bars) neurons and their respective controls (black bars). ^*^p ≤ 0.05, ^**^p ≤ 0.01, n.s. = not significant.

Most prior studies of neurons lacking Pten have reported increases in miniature EPSC amplitude (Luikart et al., 2011; Weston et al., 2012; Xiong et al., 2012; but see, Sperow et al., 2012), while the results from Tsc1-ko neurons have been mixed (Tavazoie et al., 2005; Bateup et al., 2013). Under voltage clamp and in the presence of TTX, we recorded miniature events in glutamatergic hippocampal autaptic neurons (**Figure 4**). Pten-ko neurons had mEPSC peak amplitudes that were 25% larger than control neurons, with similar decay time constants (**Figures 4A,B**). Tsc1-ko neurons’ mEPSC amplitude and decay time constants, however, were not different from control (**Figures 4A,B**). We also determined the frequency of the miniature events. mEPSCs from Pten-ko neurons were not different from control, while the Tsc1-ko neurons had significantly reduced mEPSC frequency (**Figures 4C,D**). Based on our previous report on loss of Pten in granule neurons, the effects of Tsc1 loss and lack of effect of Pten loss on mini frequency likely arise from an inhibitory effect of loss on the rate constant for spontaneous vesicle release that is independent of changes in synapse number (Weston et al., 2012).

### LOSS OF Pten, BUT NOT Tsc1, INCREASES THE NUMBER OF RELEASABLE SYNAPTIC VESICLES BUT DOES NOT ALTER THE VESICULAR RELEASE PROBABILITY

AP-evoked release in Pten-ko neurons is increased 70%, while the mEPSC amplitude is increased only 25%, suggesting that increases in other parameters may also contribute to the size of the EPSC. In addition, both Pten- and Tsc1-ko neurons have increased AP width (**Figure 2C**), which can lead to increased presynaptic calcium entry and vesicular release probability. Application of a hyperosmotic solution (500 mM sucrose) to neurons causes the release of all available synaptic vesicles in a calcium-independent manner, and can be used, in combination with the charge contained in the mEPSC, to determine the number of releasable synaptic vesicles and the probability of their release in response to an action potential (Rosenmund and Stevens, 1996; Fernandez-Chacon et al., 2001; Weston et al., 2011).

**FIGURE 5:**
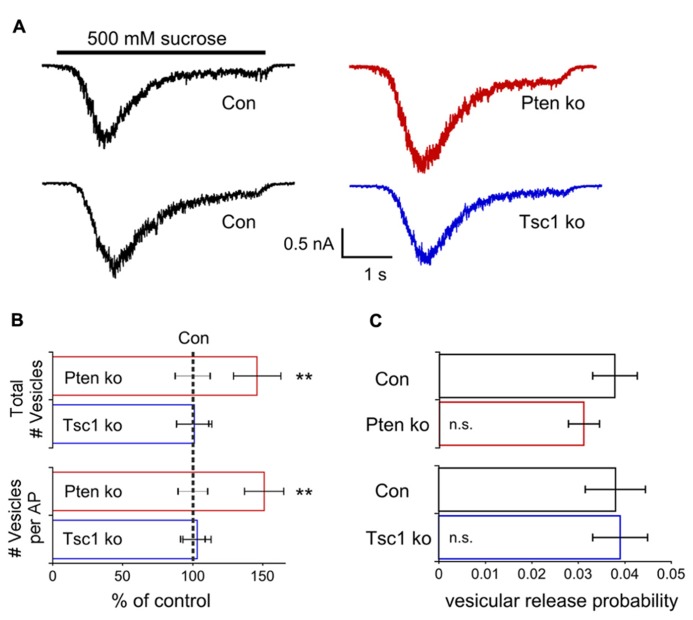
**Pten-ko neurons have increased total number of glutamatergic synaptic vesicles and released vesicles but Tsc1-ko neurons do not. (A)** Representative traces showing the response to a 4 s application of 500 mM sucrose from one Pten-ko neuron (red trace), one Tsc1-ko neuron (blue trace), and their respective controls (black traces). **(B)** Bar graph showing the total number (mean ± SEM) of glutamate-containing synaptic vesicles available for release in Pten-ko (top red bar) and Tsc1-ko (top blue bar) neurons relative to their respective controls (black dashed line), as well as the average number (mean ± SEM) of glutamate-containing synaptic vesicles released in response to an AP in Pten-ko (bottom red bar) and Tsc1-ko (bottom blue bar) neurons relative to their respective controls (black dashed line). **(C)** Bar graph showing the vesicular release probability (mean ± SEM) of Pten-ko (red bar) and Tsc1-ko (blue bar) neurons relative to their respective controls (black bars). ^*^^*^p ≤ 0.01, n.s. = not significant.

In Pten-ko neurons the current response to 500 mM sucrose application was significantly increased relative to control, while in Tsc1-ko neurons it was not (**Figure 5A**). Next, for each neuron in the dataset, we calculated the total number of releasable synaptic vesicles by dividing the charge integral of the sucrose-induced current by the charge integral of the average mEPSC. Pten-ko neurons showed a 40% increase in the number of synaptic vesicles per neuron relative to control, while the number of synaptic vesicles in Tsc1-ko neurons was not different from control (**Figure 5B**, top bars). Then, by dividing the charge integral of the AP-evoked EPSC by the charge integral of the average mEPSC, we calculated the average number of synaptic vesicles released per AP (**Figure 5B**, bottom bars). Pten-ko neurons showed a 40% increase in the number of synaptic vesicles released per AP relative to control, while the number of synaptic vesicles released per AP in Tsc1-ko neurons was unchanged.

The vesicular release probability is the probability that an available vesicle can be exocytosed in response to Ca^2^^+^ influx during an action potential. This probability can be calculated as the ratio the number of vesicles released per AP to the total number of vesicles available for release. In both Pten- and Tsc1-ko neurons, the vesicular release probability was not different from control, supporting the conclusion that loss of these molecules does not greatly affect the efficiency of synaptic vesicle release (**Figure 5C**). Taken together, these results suggest that, in addition to mEPSC size, either an increased synapse number or number of vesicles per synapse may contribute to the EPSC increase seen with loss of Pten. However, these alterations are apparently absent in Tsc-ko neurons.

### LOSS OF Pten OR Tsc1 SHOWS SIMILAR EFFECTS ON DENDRITIC MORPHOLOGY BUT DIFFERENT EFFECTS ON SYNAPSE NUMBER

**FIGURE 6:**
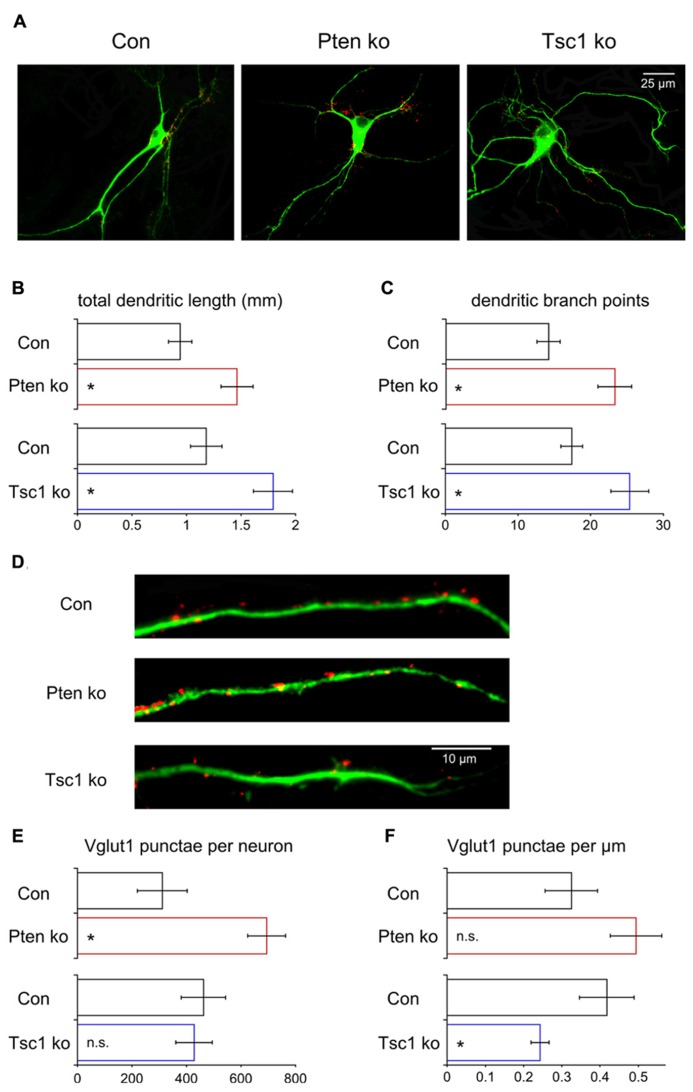
**Loss of Pten or Tsc1 has similar effects on dendrite growth but not on synapse number. (A)** Representative images showing immunofluorescence from Map2 (green) and Vglut1 (red) of control, Pten-ko and Tsc1-ko neurons. **(B)** Bar graph showing total dendrite length (mean ± SEM) of Pten-ko (red bars) and Tsc1-ko (blue bars) neurons and their respective controls (black bars). **(C)** Bar graph showing number of dendritic branch points (mean ± SEM) of Pten-ko (red bars) and Tsc1-ko (blue bars) neurons and their respective controls (black bars). **(D)** Representative images showing immunofluorescence of Map2 (green) and Vglut1 (red) on the dendrites of control, Pten-ko and Tsc1-ko neurons. **(E)** Bar graph showing total number of Vglut1 punctae (mean ± SEM) per Pten-ko (red bars) and Tsc1-ko (blue bars) neuron and their respective controls (black bars). **(F)** Bar graph showing number of Vglut1 punctae per micron (mean ± SEM) from Pten-ko (red bars) and Tsc1-ko (blue bars) neurons and their respective controls (black bars). ^*^p ≤ 0.05, n.s. = not significant.

Our electrophysiological analysis of Pten- and Tsc1-ko neurons showed that there was an increase in the number of releasable synaptic vesicles in Pten-ko neurons but not in Tsc1-ko neurons. The increase in the Pten-ko could be due to two non-exclusive possibilities: (1) An increase in the number of synaptic vesicles per synapse, or (2) an increase in the total number of synapses. To test this we immunostained our single neuron cultures for Vglut1, the synaptic vesicle protein that loads glutamate into vesicles and whose punctate staining pattern corresponds to excitatory synapses (Takamori et al., 2000; De Gois et al., 2006; Gabellec et al., 2007), and Map2 to visualize dendrites. We then imaged the neurons and reconstructed the dendritic trees using Neurolucida software and counted the number of VGlut1 positive punctae along the dendrites (**Figure 6A**). The total length of the dendrites was significantly increased in both mutants (**Figures 6A,B**), as was the number of branch points (**Figure 6C**). Control experiments showed that Map2 intensity in the soma was not elevated in Pten- or Tsc1-ko neurons relative to control (97 ± 3% of control for Pten-ko, 95 ± 5% of control for Tsc1-ko) suggesting that the changes in dendrite morphology were not due to a general increase on Map2 levels due to mTOR activation. Next, we counted the number of Vglut1-positive punctae per neuron. Pten-ko neurons had a twofold increase in the number of Vglut1 punctae, while Tsc1-ko neurons were unchanged compared to control (**Figures 6D,E**). Next, on a per neuron basis, we calculated the density of Vglut1 punctae. Pten-ko neurons had a non-significant increase in the density of Vglut1 punctae, while Tsc1-ko neurons had a significant decrease in the density of Vglut1 punctae (**Figures 6D,F**). Thus, Pten-ko neurons increase the number of their synaptic contacts in parallel with their increase in dendritic length and maintain synapse density. Tsc1-ko neurons, while also having a greater dendritic length, do not increase the number of synapses, resulting in a decrease in the overall density. Thus, loss of Pten and Tsc1 caused similar changes in the basic dendritic structure of single neurons, but the increased dendritic tree size is not sufficient to drive increases in excitatory synapse formation or neurotransmission, at least in Tsc1-ko neurons.

### LOSS OF Pten OR Tsc1 HAS OPPOSITE EFFECTS ON GABAergic NEUROTRANSMISSION

**FIGURE 7:**
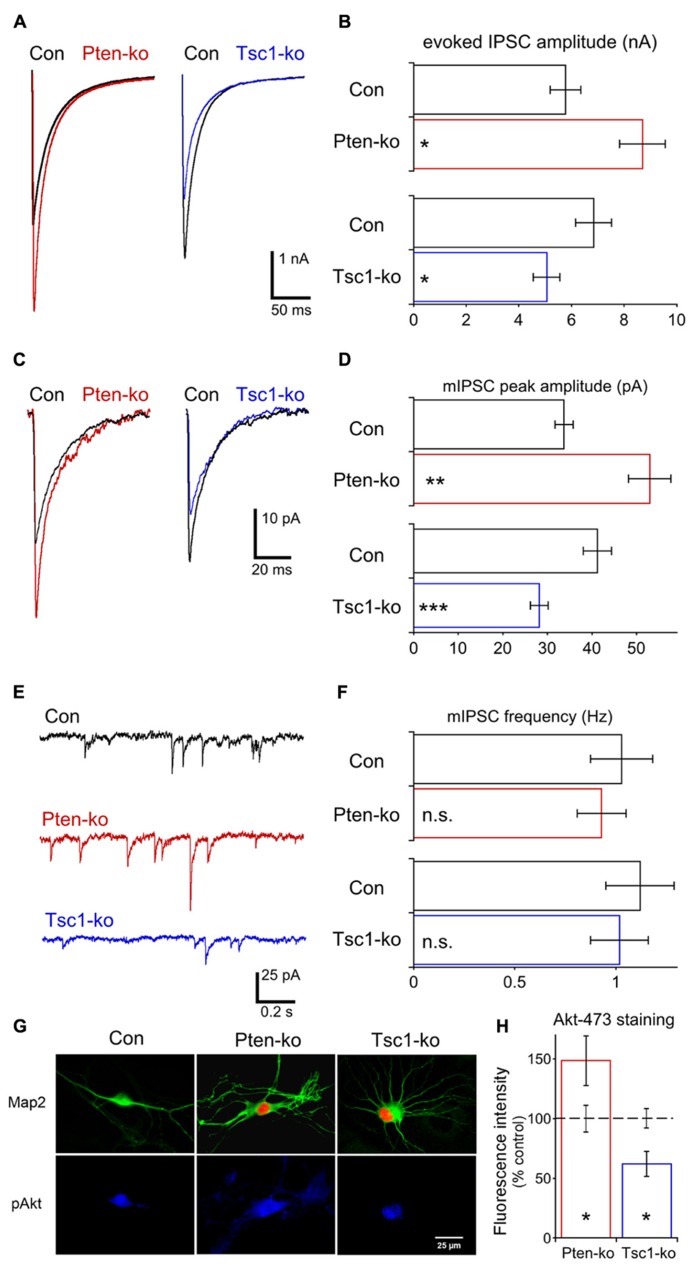
**Loss of Pten or Tsc1 has opposite effects on GABAergic synaptic transmission in hippocampal GABAergic neurons. (A)** Representative responses of voltage-clamped Pten-ko (red trace) and Tsc1-ko (blue traces) neurons and their controls (overlaid black traces) to a 2 ms depolarization to 0 mV from -70 mV. **(B)** Bar graph showing AP-evoked IPSC peak amplitudes (mean ± SEM) of Pten-ko (red bars) and Tsc1-ko (blue bars) neurons and their respective controls (black bars). **(C)** Representative traces showing the average of mIPSCs collected from one Pten-ko neuron (red trace), one Tsc1-ko neuron (blue trace) and their respective controls (overlaid black traces). **(D)** Bar graph showing mIPSC peak amplitudes (mean ± SEM) of Pten-ko (red bars) and Tsc1-ko (blue bars) neurons and their respective controls (black bars) **(E)** Representative traces showing control (black trace), Pten-ko (red trace), and Tsc1-ko (blue trace) miniature postsynaptic current activity from hippocampal GABAergic neurons. **(F)** Bar graph showing mIPSC frequencies (mean ± SEM) of Pten-ko (red bars) and Tsc1-ko (blue bars) neurons and their respective controls (black bars). **(G)** Representative images showing immunofluorescence from Map2 (green) and p-Akt S473 (blue) from control (left column), Pten-ko (middle column) and Tsc1-ko (right column) neurons. Red fluorescence in Pten- and Tsc1-ko neurons is from the Cre-RFP fusion protein. **(H)** Intensity values (mean ± SEM) for pAkt measured from Pten-ko (red bar) and Tsc1-ko (blue bar) relative to their respective controls (black dashed line). ^*^p ≤ 0.05, ^*^^*^p ≤ 0.01, ^***^p ≤ 0.001, n.s. = not significant.

Glutamatergic transmission was normal in Tsc1-ko neurons, and the current clamp analysis did not show any evidence for intrinsic hyperexcitability (**Figure 2**), however, network activity in Tsc1 deficient brains and cultures shows signs of hyperexcitability. We therefore tested whether the Pten and Tsc-ko neurons had any alterations in GABAergic transmission. We performed a whole cell voltage clamp analysis of single hippocampal GABAergic Pten- and Tsc1-ko neurons. A high-chloride intracellular solution was used so that GABA-gated chloride conductances elicited inward currents, and application of bicuculline verified the synaptic events were GABAergic. First, we recorded AP-evoked GABAergic synaptic responses in Pten- and Tsc1-ko neurons (**Figures 7A,B**). In Pten-ko neurons, the mean peak amplitude of IPSCs was increased by 50%, while in Tsc1-ko neurons the mean peak amplitude of IPSCs was decreased by 25% (**Figures 7A,B**). Next, we recorded miniature synaptic transmission. In Pten-ko neurons, the mean peak amplitude of mIPSCs was increased by 50%, but the frequency was unchanged (**Figures 7C–F**). In Tsc1-ko neurons, the mean peak amplitude of mIPSCs was decreased by 30% (**Figures 7C,D**), while the frequency was unchanged (**Figures 7E,F**). These changes in miniature event size indicate that the changes in AP-evoked GABAergic transmission are due to reduced mini size, and most likely altered postsynaptic GABA receptor number.

Previous reports have suggested that the ß subunit of the GABA_A_ receptor is a substrate of Akt (Wang et al., 2003; Xu et al., 2006). Akt phosphorylation of this subunit increases the amount of GABA_A_ trafficked to the postsynapse and the amplitude of miniature IPSCs (mIPSC), and mediates an increase in GABAergic transmission in response to insulin (Wang et al., 2003; Xu et al., 2006; Luscher et al., 2011). Since loss of Pten and Tsc1 have been reported to have opposite effects on Akt activation, we immunostained our single neuron cultures for phospho-Akt 473 and quantified the effects of Pten and Tsc1 loss on fluorescence levels. Loss of Pten caused a 50% increase in the intensity of pAkt473 fluorescence, while loss of Tsc1 caused a 40% decrease in the intensity of pAkt473 fluorescence (**Figures 7G,H**), suggesting that the differential effect of Pten and Tsc1 loss on Akt activity may regulate inhibitory transmission in opposite directions.

## DISCUSSION

In this study, we performed a direct head-to-head comparative analysis of neurons lacking one of two genes that are both repressors of the mTOR signaling pathway and whose loss causes epilepsy and autism in animal models and humans. We undertook this study in order to identify cellular phenotypes shared by neurons lacking *Pten* and *Tsc1*, reasoning that, since the disease phenotypes caused by loss of the two genes are similar, that the cellular phenotypes shared by the two might contribute to the organismal consequences of loss in a meaningful way. We expected, based on our previously published data on Pten, and on the hypothesis that alterations in the balance of excitation and inhibition in the brain may contribute to the development of both epilepsy and autism, that loss of either gene would lead to a direct increase in excitatory synaptic transmission.

In contrast to our original expectation, we found that only loss of Pten caused a direct increase in excitatory synaptic transmission, and that loss of Tsc1 had no measurable effect. Loss of Tsc1 did, however, cause a reduction in inhibitory synaptic transmission, an effect whose consequences may be functionally equivalent to an increase in excitatory transmission, especially in the context of the excitation-inhibition balance in a neuronal circuit. Other cellular effects of Pten and Tsc1 loss, such as the effects on neuronal growth, were similar, suggesting that these changes are likely important factors contributing to the disease phenotypes common to loss of Tsc1 or Pten.

### SIMILARITIES OF Pten AND Tsc1 LOSS

Many of the molecular and cellular effects of Pten and Tsc1 loss were quantitatively similar. The majority of these changes have been described previously, including: (1) the increases in somatic and dendritic growth, (2) the alterations in membrane resistance and capacitance, and (3) the increases in levels of mTORC1 effectors pS6 and p4E-BP. Although our data showing changes in these parameters are not novel for loss of either gene, our study is the first to compare them side-by-side in the same experimental conditions. This is important for two reasons. First, the repeatability of the phenotypes gives further credence to the notion that they are direct consequences of Pten/Tsc1 loss and mTORC1 complex activation, and second, the side-by-side comparison shows that the magnitude of the changes caused by loss of either gene are comparable.

We also found that rapamycin treatment for 72 h caused a severe reduction in pS6 levels and normalized the soma area in both Pten- and Tsc1-ko neurons, although it was ineffective at reducing p4E-BP. The ability of rapamycin to inhibit the excessive S6 phosphorylation and cell growth in neurons caused by Pten and Tsc1 loss is well established, and there is also evidence that it can prevent increases in synaptic transmission caused by Pten loss (Weston et al., 2012; Xiong et al., 2012). The lack of an effect of long-term (>6 h) rapamycin on phosphorylation of 4E-BP and translation was previously shown to be cell-type dependent (Choo et al., 2008). Our data here showing that rapamycin does not decrease p4E-BP levels in hippocampal neurons caused by Pten or Tsc1 loss is therefore important to consider when interpreting the effects of rapamycin on neurons, neural circuits and the whole brain.

Although previously reported for loss of Pten (Weston et al., 2012), we show here that loss of Tsc1 also leads to a decrease in the amplitude and increase in the half-width of action potentials in hippocampal neurons. Interestingly, a recent study of neurons lacking FMRP also reported an increase in AP duration (Deng et al., 2013). Loss of FMRP causes Fragile X syndrome, a disorder in which autism and epilepsy are frequently present, suggesting that altered presynaptic calcium influx due to changes in action potential shape could contribute to these phenotypes. We did not, however, detect any changes in the probability of synaptic vesicle release in Pten- and Tsc1-ko neurons, which would be expected in response to changes in presynaptic calcium influx. In addition, two previous studies in Tsc1-lacking neurons reported either no change (Bateup et al., 2013), or an increase in the AP amplitude and apparent decrease in the half-width of neurons lacking Tsc1 (Normand et al., 2013). Thus, the physiological impact of the changes in AP shape on the alterations in synaptic transmission that we observed remains unclear at this time.

Despite the fact that we found reduced input resistance and no change in the AP voltage threshold, we did not detect an increase in the amount of injected current required to elicit an AP (**Figure 2**; **Table 1**). There are at least two possibilities for this. First, the magnitudes of the changes we found in *R*_m_ were not large (20–25% decrease), and there was a small but non-significant shift in the AP threshold toward more hyperpolarized values. Thus, any change in threshold current due to Pten or Tsc1 loss may be simply beyond our detection limit. Second, it is also possible that loss of Pten or Tsc1 causes changes in voltage gated conductances activated by the depolarization that compensate for the reduced R_m_ to normalize the current threshold. Regardless of the effects of Pten or Tsc1 loss on current threshold, our data do not support the position that the hyperexcitability demonstrated by Pten- or Tsc1-deficient networks is due to intrinsic increases in the excitability of glutamatergic neurons.

### DIFFERENCES OF Pten AND Tsc1 LOSS: SYNAPTIC TRANSMISSION

Despite the similarities of Pten and Tsc1/2, especially in their influence on mTORC1 activity, we show here that their direct effects on synaptic transmission in hippocampal neurons are strikingly different. We show that loss of Pten causes a large increase in AP-evoked glutamatergic transmission due to the combined effect on the mEPSC size and the number of synapses, while loss of Tsc1 causes no change. For loss of Pten, this increase in excitatory transmission has support from several studies in cortical (Xiong et al., 2012), dentate granule (Luikart et al., 2011; Weston et al., 2012; Takeuchi et al., 2013), and now other hippocampal neuron types (CA1 and CA3, this study), although one previous study reported a decrease in CA1 fEPSPs (Fraser et al., 2008). While initial reports of Tsc1 loss in hippocampal pyramidal neurons suggested increased AP-evoked glutamatergic transmission (Tavazoie et al., 2005; Bateup et al., 2011), more recent studies, even from the same group, have not shown an increase in hippocampal (Bateup et al., 2013) or Purkinje neurons (Tsai et al., 2012). Our results support the more recent findings suggesting that loss of Tsc1 does not increase evoked glutamatergic transmission.

We found that neurons lacking Pten have increased mIPSC amplitude while those lacking Tsc1 have decreased mIPSC amplitude, and that the changes in mIPSC amplitude are mirrored by changes in AP-evoked IPSCs. The regulation of inhibitory synaptic transmission by mTOR has been studied far less than the regulation of excitatory transmission, but our results are supported by two previous studies. Striatal neurons lacking Pten showed increased mIPSC and AP-evoked IPSC amplitudes (Weston et al., 2012) and hippocampal pyramidal neurons lacking Tsc1 showed decreased mIPSC and evoked IPSC amplitudes (Bateup et al., 2013). However, shRNA-mediated knockdown of Pten has been reported not to produce a significant increase in IPSC amplitude in dentate granule neurons (Luikart et al., 2011).

*In vivo*, the parameters that regulate the synaptic input and output of a neuron are determined by intrinsic factors, such as its genetic program, and extrinsic factors, such as activity-dependent mechanisms. In the autapic neuron preparation that we use here, the synaptic properties are largely determined by intrinsic factors, as autaptic neurons do not exhibit classic mechanisms of plasticity such as LTP or homeostatic scaling (Chang et al., 2014). Although many of the changes in synaptic transmission due to Pten and Tsc1 loss in this work are similar to those seen in acute slice, some effects seen only in slice may be due to network-dependent effects, while others may be masked in slice due to network compensation. Understanding whether certain consequences of Pten or Tsc1 loss are cell autonomous or network dependent is important, however, and the comparison of the autaptic culture to acute slice and even *in vivo* data, if available, can help answer these questions.

### DIFFERENCES OF Pten AND Tsc1 LOSS: MOLECULAR EFFECTS

In this report we show, as others previously, that the phosphorylation of Akt at S473 is increased by Pten loss and decreased by Tsc1 loss in the brain (Groszer et al., 2001; Kwon et al., 2001; Meikle et al., 2008; Ljungberg et al., 2009; Carson et al., 2012). Altered levels of phospho-Akt S473 would be expected to increase or decrease Akt activity toward its substrates (Sarbassov et al., 2005b). Previous reports have shown that knockdown of Akt with siRNA decreased glutamatergic synapse formation (Majumdar et al., 2011), and activating PI3K (analogous to loss of Pten in its effect on Akt phosphorylation) has been shown to increase the exocytosis of AMPA receptors and increase mEPSC amplitude (Man et al., 2003). While pharmacological inhibition of PI3K (analogous to loss of Tsc1 in effect on Akt phosphorylation) does not affect AMPA receptor insertion at baseline conditions (Man et al., 2003), it does block the insertion of AMPA receptors in response to synaptic stimulation and homeostatic compensation (Qin et al., 2005; Hou et al., 2008; Shehata et al., 2012). Thus, it is possible that activation of Akt in Pten-ko neurons leads to enhanced trafficking of AMPA receptor subunits to the synaptic membrane, which could be a critical step in increased synapse formation in these cells.

While the list of potential Akt substrates is long and the cellular effects of altered Akt activity are likely to be complex, the ß subunit of the GABA_A_ receptor stands out as particularly relevant to the effects on GABAergic transmission we report here. Since Akt phosphorylation of this subunit increases the amount of GABA_A_ receptor trafficked to the synapse and the amplitude of mIPSCs (Wang et al., 2003; Xu et al., 2006; Luscher et al., 2011), it is possible that the changes in inhibitory synaptic transmission in response to Pten and Tsc1 loss are results of the effects on Akt activity.

While the differences in Akt activity may contribute to the effects on synaptic transmission, other factors are likely to be involved. The ability of Pten loss to increase excitatory neurotransmission could be independent of downstream mTOR signaling, and be due instead to Pten activity toward substrates other than PIP_3_. This hypothesis is attractive in its simplicity, however, the ability of rapamycin to prevent the increases in EPSCs strongly argues against this contention (Weston et al., 2012; Xiong et al., 2012). Nonetheless, Pten’s protein phosphatase activity alone appears to be sufficient to decrease the number of dendritic spines in slice culture (Zhang et al., 2012), and Pten has been reported to bind to PSD-95 in response to NMDA receptor activation (Jurado et al., 2010), suggesting that alternative, postsynaptic targets may exist.

Taken together, our results suggest that excessive mTORC1 activity caused by loss of either Pten or Tsc1 is sufficient to drive increases in neuronal growth, but not increases in synapse formation and glutamatergic neurotransmission. However, our data also suggest that enhanced excitatory transmission may not be a necessary prerequisite to produce the disease phenotypes associated with hyperactivation of mTOR signaling, since loss of Tsc1-mediated suppression of synaptic inhibition may contribute to an overall excitatory-inhibitory imbalance.

## MATERIALS AND METHODS

### MICE AND CELL CULTURE

Animal housing and use were in compliance with the National Institutes of Health (NIH) Guidelines for the Care and Use of Laboratory Animals and were approved by the institutional animal care committee at Baylor College of Medicine. Experiments utilized Pten^tm1Hwu^/J mice that possess loxP sites on either side of exon 5 of the PTEN gene, and Tsc1^tm1Djk^/J mice that possess loxP sites preceding exon 17 and downstream of exon 18 (The Jackson Laboratory). The mice were backcrossed to C57BL/6 wild-type mice for at least six generations to ensure comparable backgrounds.

Microisland cultures of P0 hippocampal neurons were prepared according to published procedures (Reim et al., 2001; Weston et al., 2011, 2012). Microislands were made by coating collagen (0.7 mg ml^-^^1^) and poly(D-lysine) (0.1 mg ml^-^^1^) on coverslips with a custom-built stamp to achieve uniform size (200 μm diameter). Astrocytes were grown on microislands for 1 week before plating of neurons. The hippocampus was removed from P0 mice of either sex. Neurons were then digested with papain (Worthington, Lakewood, NJ, USA) and plated on astrocytes derived from wild-type neonatal cortex tissue at a density of 2000–3000 neurons per 35 mm dish and grown in a chemically defined medium (Neurobasal-A medium supplemented with Glutamax and B-27; Invitrogen). Under these conditions, a single neuron on an astrocyte microisland forms recurrent synapses (autapses) (Bekkers and Stevens, 1991).

All experiments were performed on two to four independent cultures of neurons from either Pten^loxP/loxP^ or Tsc1^loxP/loxP^ mice infected with a lentivirus expressing a Cre recombinase-RFP fusion protein with a nuclear localization signal under control of the synapsin promoter 18–24 h after plating. For all experiments, two different types of control neurons were used, either Pten^loxP/loxP^ or Tsc1^loxP/loxP^ infected with a control lentivirus expressing RFP at the same multiplicity of infection, or wild-type littermates of the experimental animals infected with the Cre recombinase-RFP fusion protein expressing lentivirus in the same amount. The values obtained from the two different control groups were not significantly different for any parameter tested and were therefore combined (see **Table TA1** in Appendix).

### ELECTROPHYSIOLOGY

Standard extracellular solution contained the following (in mM): 140 NaCl, 2.4 KCl, 10 HEPES, 10 glucose, 4 MgCl2, and 2 CaCl2, pH 7.3 (305 mOsm). Internal solution contained the following: 136 mM KCl, 17.8 mM HEPES, 1 mM EGTA, 0.6 mM MgCl2, 4 mM ATP, 0.3 mM GTP, 12 mM creatine phosphate, and 50 U/ml phosphocreatine kinase. These concentrations set the chloride reversal potential high enough that GABA receptor mediated synaptic responses resulted in an inward current. All experiments were performed at room temperature (23–24°C). Whole-cell recordings were performed on neurons from control and experimental groups in parallel on the same day *in vitro* (day 9–14 *in vitro*). Only neurons with visually identified expression of fluorescent reporters were targeted for patching.

For voltage clamp experiments, neurons were held at -70 mV unless noted. Action potential-evoked EPSCs or IPSCs were triggered by a 2 ms somatic depolarization to 0 mV. The shape of the evoked response and antagonists [either 3 mM kynurenic acid (Tocris Bioscience) or 20 μM bicuculline (Tocris Bioscience)] were applied to verify glutamatergic or GABAergic identities. Neurons were stimulated at 0.2 Hz (for EPSCs) or 0.1 Hz (for IPSCs) in standard external solution to measure basal evoked synaptic responses. The number of releasable synaptic vesicles of each neuron was determined by measuring the charge transfer of the transient synaptic current induced by a 4 s application of hypertonic sucrose solution directly onto the neuron and then dividing the sucrose charge by the charge of the average miniature event from the same neuron (Rosenmund and Stevens, 1996). To obtain *P*_vr_, the basal evoked synaptic responses and the response to the hypertonic sucrose solution were recorded successively from the same neuron. The evoked response was integrated to calculate the charge transfer. *P*_vr_ was calculated as the ratio of evoked response charge to RRP charge (Reim et al., 2001; Weston et al., 2011). For current clamp experiments, bias current was injected to achieve a resting membrane potential of -70 mV and kynurenic acid or bicuculline were applied to block synaptic responses. Input resistance and membrane time constant were calculated from the steady state and charging transient, respectively, of voltage responses to 0.5 s, 25 pA hyperpolarizing current steps. Membrane capacitance was calculated by dividing the time constant by the input resistance. Action potentials (APs) were evoked with 0.5 s 25 pA depolarizing current steps. AP threshold was defined as *V*_m_ at the inflection point of the rising phase of the AP. AP amplitude was defined as the difference in *V*_m_ between the peak and threshold.

Data were analyzed offline with AxoGraph X 1.0 (AxoGraph Scientific, Sydney, Australia) and KaleidaGraph (Synergy Software, Reading, PA, USA). Values for analysis were always pooled from at least two independent cultures. Normality was tested by the Kolmogorov–Smirnov test. Statistical significances were tested by using Student’s *t* test, or the non-parametric Mann–Whitney test. One-way ANOVA followed SNK post-test was used when analyzing three groups.

### MINIATURE EVENT ANALYSIS

Miniature synaptic potentials were recorded for 60–80 s with either 3 mM kynurenic acid or 20 μM bicuculline applied for 2 s of every 10 s for background noise subtraction and to verify the identity (glutamatergic or GABAergic) of the neuron under study. For some neurons, 500 nM tetrodotoxin (TTX) was applied, however, there was no difference in the amplitude or frequency of events with or without TTX (Weston et al., 2012). For each cell, data were filtered at 1 kHz and analyzed using template-based miniature event detection algorithms implemented in analysis software AxoGraph X 1.0. Threshold for detection was set at three times the baseline SD from a template of 0.5 ms rise time and 3 ms decay for glutamatergic events and 0.5 and 20 ms for GABAergic events.

### VIRUS PRODUCTION

The preparation of lentiviral particles expressing Cre-RFP fusion protein or the RFP under control of the synapsin promoter was done as previously described (Lois et al., 2002). Briefly, HEK293T cells were co-transfected with 8 μg shuttle vector F(SYN) UGW-RBN bearing cDNA for cre-RFP, and the mixed helper plasmids pCMVdR8.9 and pVSV-G (5 μg each) with Fugene 6 transfection reagent (Roche Diagnostics, Indianapolis, IN, USA). After 48 h the cell culture supernatant was collected and cell debris was removed by filtration with a 0.45 μm polyethersulfone membrane (Pall Life Sciences, Ann Arbor, MI, USA). Aliquots of the filtrate were flash frozen in liquid nitrogen and stored at –80°C. Estimation of the titer was done on mass cultures of wild-type hippocampal neurons. For infection of the neurons for experiments 100 μl of the viral solution (0.9–1.8 × 10^6^ i.u./ml) was used 18–24 h after plating.

### IMMUNOCYTOCHEMISTRY AND ANALYSIS

After electrophysiology experiments were completed, neurons were rinsed with PBS and fixed in 4% paraformaldehyde for 30 min, then washed three times with PBS again. Samples were incubated in blocking buffer (5% normal horse serum, 0.1% Triton X-100 in PBS) at room temperature for 1 h. Primary antibodies diluted in blocking buffer were applied overnight at 4°C. The following antibody dilutions were used: rabbit anti-phospho-S6 Ser 240/244 (1: 1,000, Cell Signaling Technology, Danvers, MA, USA), mouse anti-MAP2 (1:2000, Millipore/Chemicon, Temecula, CA, USA), rabbit anti-MAP2 (1:2000, Millipore/ Chemicon) rabbit anti-phospho-Akt (Ser473; 1: 1,000, Cell Signaling Technology), rabbit anti-phospho-4E-BP (Thr37/46; 1: 1,000, Cell Signaling Technology) guinea pig anti-VGLUT1 (1: 5,000, Synaptic Systems, Goettingen, Germany). After three washes for 5 min in PBS, secondary goat antibodies coupled to/conjugated with Alexa 488, 555, or 647 (Invitrogen/Molecular Probes, Eugene, OR, USA) were diluted 1:1000 in blocking buffer and applied for 1 h at room temperature. All images were obtained using Zeiss AxioImager M1 system with 40× Zeiss objectives and AxioVision software.

For neuron reconstruction and analysis, images were acquired using equal exposure times and processed using the US National Institutes of Health ImageJ (). A threshold macro was applied to the VGLUT channel to identify synapses. For cell body and dendrite tracing, the contrast of the images was increased so that the edges of the soma and the tips of dendrites were clearly visible to ensure the accuracy of the tracing. Neuron cell bodies, dendrites, and synapses were drawn using Neurolucida software (MicroBrightField, Williston, VT, USA). Quantification of the traced images was conducted with Neuroexplorer software (MicroBrightField). Raw values were then exported to KaleidaGraph (Synergy Software) for further analysis.

To quantify immunofluorescence intensity of pS6, p4E-BP, and pAkt, images of Map2 and the other antigen were acquired with equal exposure times and settings for control and experimental groups, assuring that none of the pixels were saturated. The images were then opened in ImageJ and the background was subtracted. Polygon selections were drawn around the cell body excluding the nucleus using the Map2 image. The average pixel intensity of the selected area in the pS6, p4E-BP, or pAkt image was then measured for each neuron.

## Conflict of Interest Statement

The authors declare that the research was conducted in the absence of any commercial or financial relationships that could be construed as a potential conflict of interest.

## APPENDIX

**Table A1:** Comparison of the two different control groups for multiple measurements.

	Pten		Tsc1	
	Wild-type, cre-rfp virus	Pten^flox/flox^, rfp only virus	Wild-type, cre-rfp virus	Tsc1^flox/flox^, rfp only virus
pS6 intensity (a.u.)	2959 ± 530 *n* = 9	3247 ± 417 *n* = 9, *p* = 0.7	3453 ± 530 *n* = 7	3780 ± 571 *n* = 10, *p* = 0.6
C_m_ (pF)	78.0 ± 6.7 *n* = 15	84.0 ± 8.1 *n* = 17, *p* = 0.6	84.6 ± 9.5 *n* = 15	74.2 ± 5.9 *n* = 18, *p* = 0.4
EPSC amp. (nA)	1.81 ± 0.27 *n* = 16	2.16 ± 0.5 *n* = 18, *p* = 0.9	1.69 ± 0.27 *n* = 17	1.8 ± 0.35 *n* = 20, *p* = 0.8
mEPSC amp. (pA)	24.1 ± 2.9 *n* = 15	25.3 ± 2.2 *n* = 17, *p* = 0.8	26.4 ± 2.8 *n* = 13	26.2 ± 1.9 *n* = 15, *p* = 0.9
RRP size (# of vesicles)	6852 ± 1573 *n* = 16	6561 ± 1684 *n* = 18, *p* = 0.7	7550 ± 1701 *n* = 17	8382 ± 2144 *n* = 20, *p* = 0.8
IPSC amp. (nA)	6.29 ± 0.6 *n* = 15	5.37 ± 1.1 *n* = 17, *p* = 0.5	6.66 ± 1.6 *n* = 17	7.00 ± 1.3 *n* = 18, *p* = 0.8
mIPSC amp. (pA)	35.7 ± 4.6 *n* = 15	34.2 ± 2.7 *n* = 13, *p* = 0.7	43.3 ± 4.8 *n* = 14	38.8 ± 4.0 *n* = 18, *p* = 0.5

*Measurements are shown as mean ± SEM; *p* value calculated with unpaired t-test or Mann–Whitney.*
